# A Microscale–Optical Interface to Examine Electric Field-Induced Cell Motility Within Whole-Eye Facsimiles

**DOI:** 10.3390/micro5010010

**Published:** 2025-02-28

**Authors:** Sakshi Koul, Luke A. Devecka, Mark C. Pierce, Maribel Vazquez

**Affiliations:** Department of Biomedical Engineering, Rutgers, The State University of New Jersey; Piscataway, NJ 08854 USA

**Keywords:** retina, transplantation, vision loss, infiltration, confocal microscopy, galvanotaxis

## Abstract

Microscale systems have been underexplored in contemporary regenerative therapies developed to treat vision loss. The pairing of in vitro cell systems with optical fluorescent imaging provides unique opportunities to examine the infiltration of donor stem cells needed for successful transplantation therapies. A parallel eye device was developed to provide electric field (EF) stimulation to guide the migration of cells within 3D eye facsimiles synthesized from different ocular biomaterials. Cell infiltration within facsimiles was rapidly resolved using confocal microscopy to eliminate dependence on the cryostat sectioning commonly used for cell study. Moreover, EF stimulated galvanotaxis of donor cells within different depths of eye facsimiles. Optical imaging provided rapid resolution of z-stack images at physiologically appropriate depths below 500 microns. This study demonstrates that paired microscale–optical systems can be developed to elucidate understudied transplantation processes and improve future outcomes in patients.

## 1. Introduction

Modern microtechnology can beget significant advancements in emerging biomedical therapies through rapid, quantitative study of complex physiological processes. A growing community has begun to bypass specialized fabrication facilities and machinery to develop micro- and mesoscale tools that readily integrate with optical microscopy to aid in the translation of novel treatments [1,2]. Such integrated systems are exceptionally well applied for regenerative therapies in the visual system, where different components of ocular physiology exhibit functional characteristic lengths below 1000 μm [3,4]. This micro/mesoscale facilitates creative and practical applications of rapid prototyping, parallelization, and optical fluorescence imaging to examine the behavior and responses of specialized cell groups in real time.

**Figure 1.**
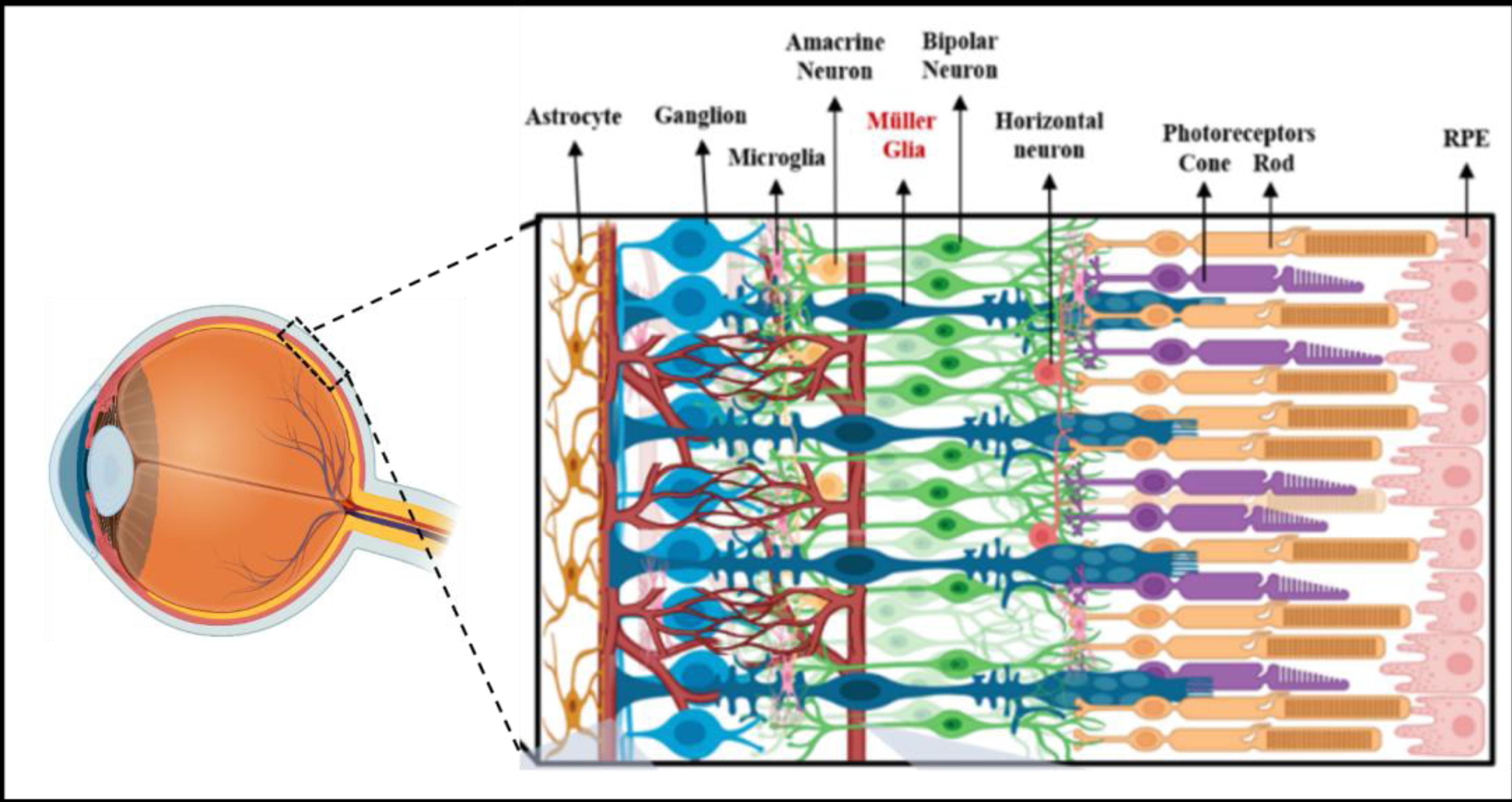
Schematic of the healthy adult retina. The human retina is a complex structure composed of specialized retinal neurons and Muller glia (MG) connected across three synaptic layers. The retinal pigment epithelium (RPE) is a single-cell layer adjacent to the rod and cone photoreceptors of the outer nuclear layer (ONL). Photoreceptors synapse with secondary neurons in the inner nuclear layer (INL), i.e., the horizontal, amacrine, and bipolar cells shown. These secondary neurons then synapse to communicate with retinal ganglion cells within the ganglion cell layer (GCL). These final projection neurons then transmit signals to the brain through the optic nerve. MG shown within the retinal thickness regulates retinal homeostasis through osmotic and ionic balance.

Vision loss in adults is an escalating global health challenge that can benefit dramatically from quantitative integrated platforms for the cellular study of emerging bioengineering treatments [5,6]. Progressive vision loss in Western countries arises most commonly from degeneration of the retina, with the incidence of disorders, such as glaucoma, diabetic retinopathy, and age-related macular degeneration, increasing dramatically in people over the age of 55 [5,6]. Promising regenerative treatments to restore vision include cell replacement therapy, where stem cells are combined with biomaterials and transplanted into the damaged retina to replace dysfunctional and apoptotic neurons [7,8], as shown in Figure 1.

In an ideal model, replacement cells re-establish vision when transplantation enables the cells to infiltrate retinal laminae and achieve functional, synaptic communication with native retinal neurons [9]. While engineers have evaluated combinations of microenvironmental cues to guide behaviors of replacement, very small numbers of stem cells migrate out of the delivery biomaterial and into retinal tissue [10–12].

The application of electric fields (EFs) to guide the directional migration of cells, known as galvanotaxis or electrotaxis, has been routinely applied in the nervous system (NS). In transcranial direct stimulation, EFs were shown to increase visual processing speed [13] and improve vision in patients with proliferative diabetic retinopathy. Non-invasive EFs have clinically delivered weak electrical currents in the eye for trans-orbital, trans-corneal, and trans-scleral stimulation and demonstrated gains in visual acuity, neurotrophins, anti-apoptosis, and anti-inflammatory pathways [14]. However, few studies have applied therapeutic EFs to aid cell transplantation into the neuroretina [15–17].

Our group has been among the first to apply EFs to guide the migration of replacement cells within a system of microfluidic channels [18,19] and subsequently within spherical ocular biomaterials [20,21]. However, further advancing the study of EFs for cellular transplantation requires the development of experimental platforms that allow for 3D biomaterial formation, the introduction of replacement cells, the application of controlled EFs, and the measurement of cellular migration with minimal manipulation. Current approaches rely on time-intensive cryostat sectioning of 3D specimens to evaluate EF-stimulated changes in cell infiltration. While sectioning protocols are routine for cell analyses across explanted retina [22], microdevices able to apply customized fields paired with rapid, in situ, non-destructive imaging will significantly advance the study of replacement cell infiltration in cell replacement therapy.

Confocal optical microscopy is a widely used technique able to provide sub-cellular imaging at discrete depths within intact 3D volumes, without the need for physical sectioning [23]. Fluorescent labeling enables cellular tracking within this volume, enabling image processing algorithms to directly measure cellular migration within the intact specimen. Importantly, confocal microscopy has been translated from the benchtop into in vivo clinical applications for imaging organs including skin, gastrointestinal tissues, and the eye [24,25]. This provides the potential for this imaging modality to accompany cell replacement therapies in pre-clinical studies and routine patient care for assessment of treatment efficacy.

The current study has developed a parallel eye device that enables visualization of multiple eye facsimiles within a configuration similar to 48-well plates using confocal microscopy. The paired platform facilitates live imaging of replacement cells that infiltrate into eye facsimiles in response to EFs. Moreover, the optical interface performs rapid, non-destructive, and depth-resolved analysis of changes in cell penetration depth as a function of applied EF. These findings highlight a cooperative system that can be adopted by researchers to develop migration-targeted strategies for regenerative therapies in the retina and nervous system more broadly.

## 2. Materials and Methods

### 2.1. Cultured Cell Models

Replacement cells were modeled using commercial retinal progenitor cells (R28; Cat. No. EUR201, Kerafest, Inc., Boston, MA, USA), which are derived from a post-natal day-six rat retinal culture and widely used for both in vivo and in vitro testing [26]. Cells were maintained in media comprising Dulbecco’s Modified Eagle Medium (DMEM; Cat No. 11885084 Thermo Fisher Scientific, Waltham, MA, USA), 10% Fetal Bovine Serum (FBS; Cat. No. 26140, Thermo Fisher Scientific, Waltham, MA, USA), 1% 100*×* Modified Eagle Medium (MEM; Cat. No. 11140050, Thermo Fischer Scientific, Waltham, MA, USA), and 1% 100 mg/mL penicillin–streptomycin (Cat. No. 15070063, Thermo Fisher Scientific, Waltham, MA, USA). Cells were passaged at weekly intervals to maintain 80*–*95% confluency in T-75 tissue culture flasks and maintained within a biological incubator at 37 °C and 5% CO_2_. Cells in the flask were washed once with Dulbecco’s phosphate-buffered saline (1 × DPBS; Cat. No. 21–031-CV, VWR, Radnor, PA, USA) and then detached from flask surfaces using trypsin solution (Cat. No 25200072, Thermo Fisher Scientific, Waltham, MA, USA). The trypsin was neutralized with FBS, and the resulting cell solution was centrifuged at 1500 rpm for three minutes. The supernatant was then removed, and the pellet was resuspended in fresh media. Cells were replated in flasks at an approximate density of 2.1 × 10^6^ cells per mL. Fluorescent labeling of cells for imaging used either proflavine or Calcein AM (Cat. No. P2508, Sigma-Aldrich, St. Louis, MO, USA), which is a green, fluorescent stain that intercalates DNA to allow visualization of cell nuclei in both live and dead cells [27], and was prepared in a 0.01% *w*/*v* solution in PBS. Calcein AM is a green, fluorescent stain that stains the cytoplasm of live cells and fluoresces through enzymatic esterase activity [28]. Calcein AM was prepared by reconstituting Calcein AM (Cat. No. C3100MP, Invitrogen, Thermo Fisher Scientific, Waltham, MA, USA) with dimethyl sulfoxide (DMSO; Cat. No. D8418, Sigma-Aldrich, St. Louis, MO, USA) at 1 mg/mL.

### 2.2. Biomaterial Facsimiles

Eye facsimiles were produced to model enucleated rat eyes (~2.5 mm diameter) for prototype testing. Facsimiles were synthesized using separate solutions of gelatin, collagen, and sodium alginate. Gelatin-based facsimiles were synthesized using gelatin powder (Cat. No. G2500–500G, Sigma-Aldrich, St. Louis, MO, USA) dissolved in deionized water using a magnetic, stirring hot plate set at 400 rpm and 40 °C for thirty minutes. The gelatin solution was allowed to cool in spherical molds of 1 mm diameter and carefully removed.

Collagen facsimiles were synthesized using 2% 1M N-2-hydroxyethylpiperazine-N-2-ethane sulfonic acid (HEPES (1M), Cat. No. 15630080, Thermo Fischer Scientific, Waltham, MA, USA), 3.6% 0.15N sodium hydroxide (Cat. No. 1310–73-2, Sigma-Aldrich, St. Louis, MO, USA), 10% 10 × phosphate-buffered saline (10X PBS, Cat. No. 70011044, Thermo Fischer Scientific, Waltham, MA, USA), 15.7% 1X PBS (1X PBS, Cat. No. 20012027, Thermo Fischer Scientific, Waltham, MA, USA), and 67.7% 3.75 mg/mL Collagen-I (Collagen Type I, Calf Skin, Lyophilized, Cat. No. C857, Elastin Products Company, Owensville, MO, USA) added in that order. The collagen solution was left to rest in spherical molds in a biological incubator at 37 °C for three hours and carefully removed after.

Facsimiles of sodium alginate (Cat. No. W201502, Sigma-Aldrich, St. Louis, MO, USA) were synthesized with calcium chloride (Cat. No. C4901–100G, Sigma-Aldrich, St. Louis, MO, USA) to maintain spherical 3D geometries. All facsimile solutions were separately dissolved in deionized water on a magnetic, stirring hot plate set at 400 rpm and 100 °C for 1 h. After mixing, the solution was removed from the plate and desiccated until no air bubbles were present in the solution. Then, using a 1 mL Henke-Ject syringe (Cat. No. P-155, Unites States Plastic Corp., Lima, OH, USA), the 2% alginate solution was added, dropwise, to a 5% gelling solution of calcium chloride previously dissolved in deionized water. Facsimiles were formed in the gelling solution and centrifuged at 400 rpm to facilitate complete gelation. After centrifugation, eye facsimiles were stored in serum-free DMEM and maintained at 4 °C prior to experimentation.

### 2.3. Preparation of Cell-Rich Hydrogel Layer (CRHL)

Cells in complete media were resuspended within an alginate hydrogel concentration to form a cell-rich hydrogel layer (CRHL, a 2% *w*/*v* sodium alginate solution, and a 5% *w*/*v* calcium chloride gelling solution) that models the biomaterials used for contemporary transplantation. A 50 μL solution of cells was added at a concentration of 2.1 × 10^6^ cells/mL with 100 μL of 2% *w*/*v* sodium alginate solution and 50 μL of 5% *w*/*v* calcium chloride for a total volume of 200 μL.

### 2.4. Development of Hydrogel Resistor

Hydrogel resistors were formed using sodium alginate and calcium chloride concentrations, as per eye facsimiles described above, i.e., a 2% *w*/*v* sodium alginate solution and 5% *w*/*v* calcium chloride gelling solution. Briefly, 1 mL of calcium chloride was added into each chamber of the parallel eye device, followed by 3 mL of sodium alginate solution. The device was left on the tabletop to facilitate gelation for 1 h and form alginate hydrogel cylinders. After the hydrogel was formed, it was manually sliced via scalpel and the cell-rich hydrogel layer (CRHL) was placed on its top half and allowed to cure for 10 min. Afterwards, the eye facsimile was placed on top of the CRHL and the second half of the hydrogel resistor was placed on top to close the device for electrical stimulation.

### 2.5. Device Fabrication

The support plate of the device was fabricated using a three-dimensional printer (Bambu Lab, X1 Carbon 0.2 nozzle, Austin, TX, USA) with Nylon substrate from a CAD model made in Autodesk Fusion360 (version 2.0.11894, Autodesk, San Francisco, CA, USA). Carbon fiber was selected for material strength, resistance to deformation when exposed to heat, and electrical resistivity [29,30]. Prior to testing, device surfaces were thoroughly cleaned with detergent, sprayed with 70% ethanol for sterilization, dry wiped with non-abrasive tissues (Cat. No. 34155, VWR, Radnor, PA, USA), and then sterilized under UV light for 30 min. Afterwards, the device was sealed using vacuum grease to prevent leakage.

### 2.6. Assembly of the Parallel Eye

The system is first assembled by allowing the hydrogel resistors to form upon the inner surfaces of the support plane. Once the hydrogel is cured, they are carefully removed and sliced in half using a clean razor blade. Second, the support plane is then filled with one-half of the hydrogel, and the CHRL is placed on top of it and left to cure. Third, the facsimile is added on top of the CHRL followed by the other half of the hydrogel. Finally, the device is filled with cell media, covered, and sealed with vacuum grease.

### 2.7. Electrical Simulation and Computational Model

A voltage drop of 0.3 V in direct current was applied using an NI myDAQ data acquisition device (National Instruments, Austin, TX, USA) and the NI Arbitrary Waveform Generator (National Instruments, Austin, TX, USA) to generate a maximum electric field of 100 mV/cm. These parameters were used to reflect contemporary applications of electric fields in vivo [32]. The voltage stimulation was first verified using a digital multimeter (Model 77-IV, Fluke Corporation, Everett, WA, USA) and then applied across eye facsimiles for a total of 5 min. Treated facsimiles were imaged six hours afterwards. Electrodes were formed using jumper wires of 75 mm and 50 mm in length, as used in conventional electrical circuits.

The electrical stimulation of whole-eye facsimiles relies on the principle of an electric field formed across a resistor, defined as follows:

(1) $$E=\frac{dV}{dL}$$

where E is the electric field, V is the electric potential, and L is the length of the chamber. When a uniform electric field is applied, the equation reduces to the following:

(2) $$E=\frac{\text{Δ}V}{L}$$

where ΔV is determined by measurement across the electrodes.

### 2.8. Finite Element Modeling

A finite element model (FEM; COMSOL Multiphysics 5.3a, COMSOL Inc., Burlington, MA, USA) was used to predict the electric potential distribution across eye facsimiles using an ionic solution of dextran (MW = 10 kDa, $D=4.071\times 10^{-11}{\text{m}}^{2}/\text{s}$) in place of the cell-rich hydrogel layer (CRHL). The electrical transport was determined using the mass balance equation:

(3) $$\frac{\partial c_{i}}{\partial t}+\nabla \left(-D_{i}\nabla c_{i}-z_{i}u_{m,i}Fc_{i}\nabla V+c_{i}\vec{u}\right)=R_{i}$$

where $c_{i}$ is the concentration, $D_{i}$ is the diffusion coefficient, $\vec{u}$ is the fluid velocity, F is the Faraday constant, V is the electric potential, $z_{i}$ is the charge number of the ionic species, and $u_{m,i}$ is the ionic mobility. A numerical solution for the concentration of dextran in the eye facsimile was calculated over the experimental time of t=0−60 min and plotted. An electric potential map was also generated.

### 2.9. Confocal Microscopy and Image Analysis

**Figure 2.**
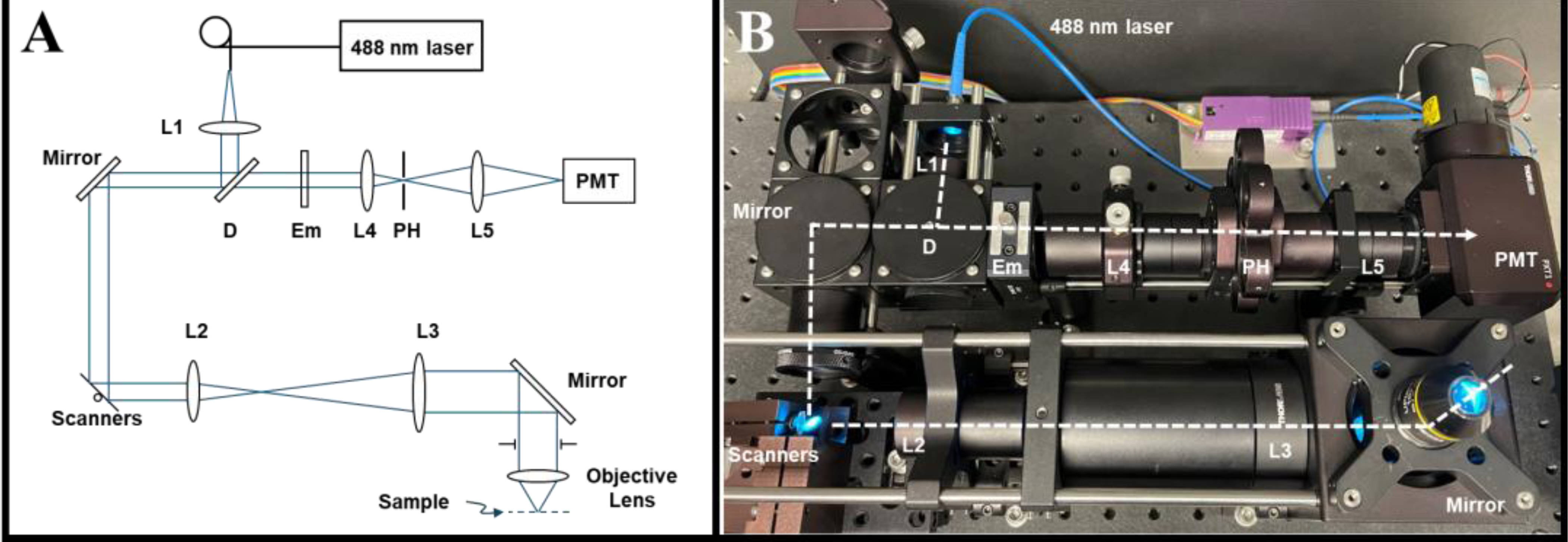
Imaging setup. (**A**) Schematic diagram of the confocal fluorescence platform. L1–4, lenses; D, dichroic mirror; Em, emission filter; PH, pinhole; PMT, photomultiplier tube. For details on components, see the text. (**B**) View of the confocal platform, showing the optical beam path (dashed line) from the illumination source fiber to the sample plane and back to the PMT detector.

A benchtop point-scanning confocal microscope was assembled for imaging eye facsimiles within 48-well plates (Figure 2). Additionally, 488 nm excitation light from a single-mode fiber-coupled laser (FiberTEC, Blue Sky Research, Milpitas, California, USA) was collimated by a 35 mm focal length lens (#49–663, Edmund Optics, Barrington, New Jersey, USA) and reflected at a dichroic mirror (Di01-R488–25×35, Semrock, Rochester, New York, USA) to a pair of close-mounted scanning galvanometer mirrors (6215H, Cambridge Technology, Bedford, Massachusetts, USA). The location between the mirrors was relayed using 50 mm and 100 mm focal length lenses (AC254–050-A, AC508–100-A, Thorlabs, Newton, New Jersey, USA) in an afocal arrangement, to the back aperture of an infinity-corrected objective lens oriented in an inverted configuration. A low-magnification objective (UPlanFL N 10x/0.3, Olympus Corporation, Hachioji-shi, Tokyo, Japan) was used to provide a comprehensive overview of the sample (950 *×* 950 μm^2^ field of view), while a high-magnification objective (LMPlanFL 50x/0.5, Olympus Corporation, Hachioji-shi, Tokyo, Japan) was used to observe localized details within the culture (190 *×* 190 μm^2^ field of view) (Supplementary Figure S1). Fluorescent emission light returned along the same path and was transmitted through the dichroic mirror and a bandpass filter (FF-01–550/88, Semrock, Rochester, New York, USA). A 40 mm focal length lens (AC254–030040-A, Thorlabs, Newton, New Jersey, USA) focused fluorescent light through a 50 μm pinhole, with a 30 mm focal length lens (LB1757-A, Thorlabs, Newton, New Jersey, USA) directing light onto a photomultiplier tube (R3896, Hamamatsu Photonics, Bridgewater, New Jersey, USA). The detector output was amplified (C9999, Hamamatsu Photonics, Bridgewater, New Jersey, USA) before being digitized at 2 MS/s (USB-6363, National Instruments, Austin, Texas, USA). Instrument control and data acquisition were accomplished with LabView software (LabVIEW 2018, National Instruments, Austin, Texas, USA) developed in-house.

Samples in standard well plates were mounted above the objective lens on a platform with manual lateral (xy) translation and a motorized z-stage (MFA-CC) and controller (ESP-301, Newport Corporation, Irvine, California, USA) to acquire confocal images at varying axial planes. Initially, the well plate containing eye facsimiles was mounted with its bottom surface at a distance exceeding the working distance of the objective lens. The z-stage was slowly lowered until the very first cells in the facsimile became visible. This axial plane was considered the outer surface of the sample, i.e., z = 0 μm. Images were then acquired at successive axial planes from z = 0 *−* 1300 μm. Cells were identified in images as bright, contiguous regions and these were manually counted within the imaged field-of-view at each depth. The total number of cells counted in each frame was then plotted against depth for both the facsimile under test (with EF) and the corresponding control (i.e., no EF).

## 3. Results

### 3.1. Fabrication of System Components and Operation

**Figure 3.**
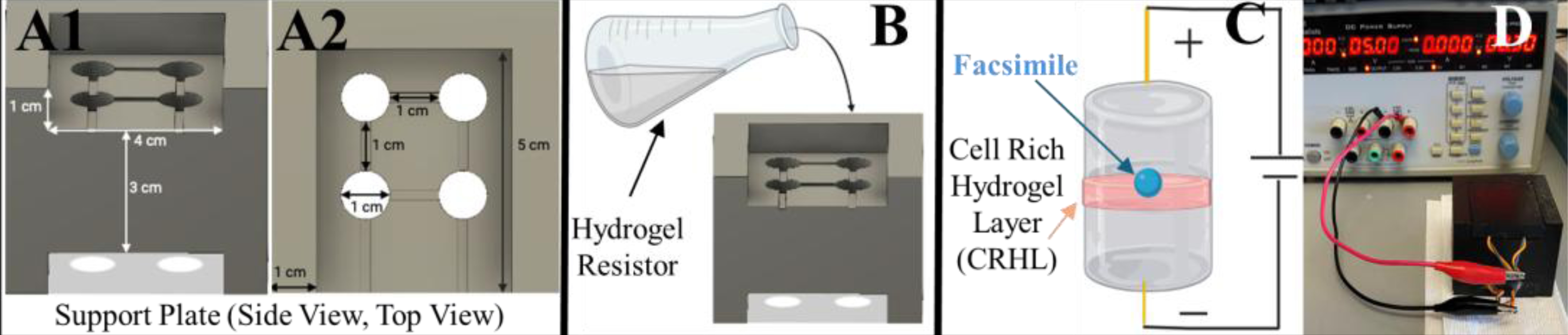
Description of the components of the parallel eye device. The system consists of three parts that work together to facilitate the application of electrical stimulation across whole-eye facsimiles and cell-rich hydrogel layers filled with replacement cells. (**A**) The support plate consists of wells with spacing and diameter comparable to conventional 48-well plates to support whole-eye facsimiles of approximate rodent size, i.e., 1 mm in diameter where (**A1**) is the side view and (**A2**) is the top view (**B**) The hydrogel resistor is poured within the wells of the support plate to gelate and produce cylinders that will maintain the eye facsimile at its center. (**C**) The hydrogel resistor is fitted with electrodes that apply an external field (EF) across its length. The resistor contains an eye facsimile and cell-rich hydrogel layer at its center to facilitate EF-induced cell infiltration within the facsimile. (**D**) An image of the final assembly is shown.

The parallel eye device consists of four components: the support plate, hydrogel resistor, biomaterial facsimile, and cell-rich hydrogel layer (CRHL). As shown in Figure 3, the support plate was designed to complement the dimensions and spacing of 48-well plates widely used for in vitro biology experiments [31,32]. The support plate measured 3 ± 0.02 cm in height, 4 ± 0.02 cm in width, and 6 ± 0.02 cm in length, as shown. The plate additionally contains four wells of 1 ± 0.02 cm in diameter that approach the configuration of commercially available 48-well plates spaced 1 ± 0.02 cm apart. These vertical wells were used to cast the hydrogel resistor, which was synthesized using alginate and calcium chloride, as described. The resistors gelate in the form of a cylinder with a 1 ± 0.02 cm diameter and a 3 ± 0.02 cm height, corresponding to the open volume within vertical wells of the support plate. The resistor holds the eye facsimile and CRHL at its center to apply external electric fields (EFs). Synthesized facsimiles exhibited a spherical volume with an average diameter of 1 ± 0.3 mm to most accurately represent the dimensions of whole-eye explants when enucleated from adult mice [33]. The CRHL, comprising replacement cells mixed within alginate, helps to fasten facsimiles within hydrogel resistors, as shown in Figure 3C. Lastly, the tip of an electrical wire is placed in the center of the hydrogel resistor to apply EF stimulation, while avoiding direct contact with cells of the CRHL.

### 3.2. Electric Simulation Validated Rapid Molecular Transport Across Parallel Eye Device

**Figure 4.**
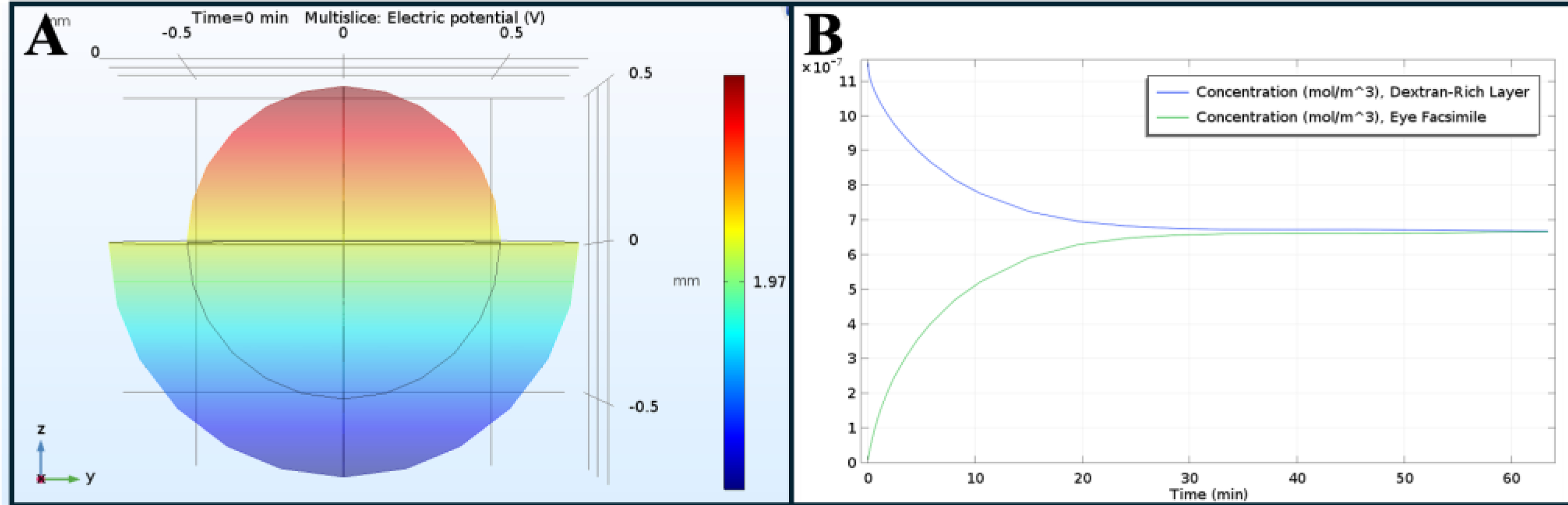
Simulation of applied electric fields (EFs) in model ionic compound. (**A**) Heat map of electric potential across eye facsimile. (**B**) Concentration release from dextran-rich layer to eye facsimile over time t = 0–60 min.

Computational analysis was subsequently used to estimate the time required for a model ionic compound to penetrate eye facsimiles. Figure 4 illustrates the diffusive transport of dextran modeled from the CRHL through the cross-section of spherical facsimiles. As shown, a voltage is generated across the facsimile that promotes the diffusion of anionic dextran, and the dextran migrates from the CRHL into the facsimile.

### 3.3. Electric Simulation Validated Rapid Molecular Transport Across the Parallel Eye Device

**Figure 5.**
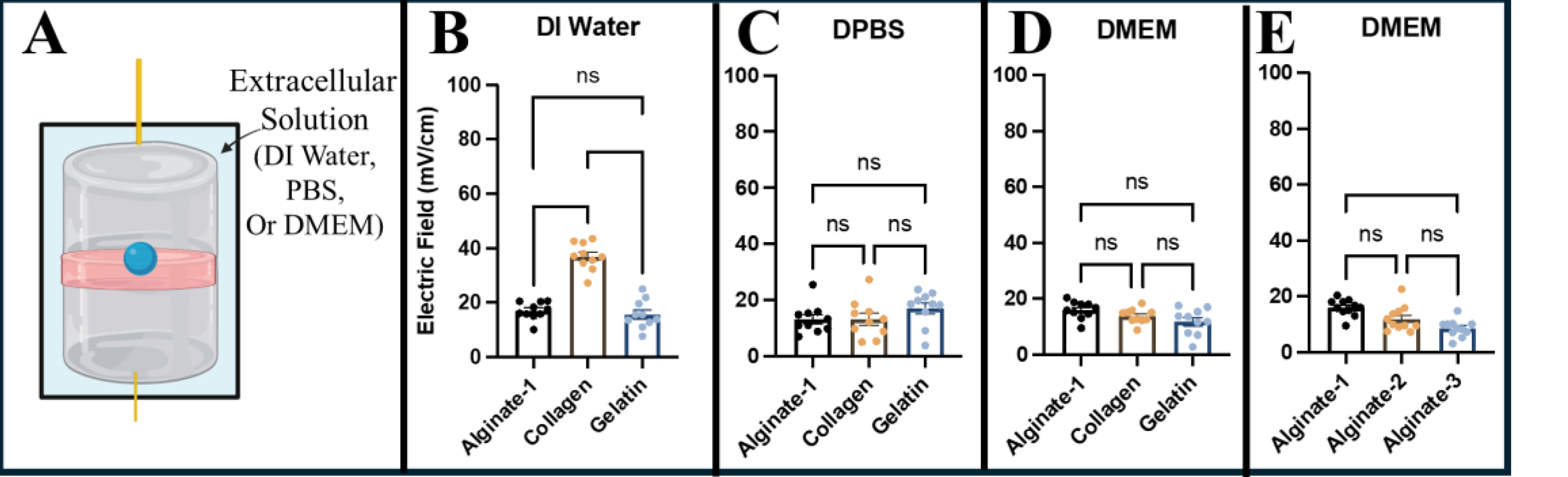
Electric field induced across eye facsimiles composed of alginate, collagen, and gelatin submerged within different biological buffers. (**A**) Schematic of the measurements performed. Average values of electric fields of facsimiles produced when stimulation is applied across (**B**) extracellular solutions (light blue) of DI water with hydrogel electrodes (gray) and CHRL (red), (**C**) Dulbecco’s phosphate-buffered saline (DPBS), and (**D**) Dulbecco’s Modified Eagle Medium (DMEM). (**E**) Average value of electric fields of alginate facsimiles (dark blue) produced across solutions of cell media when synthesized with DI water (Alginate-1), DPBS (Alginate-2), and DMEM (Alginate-3). Statistical significance is denoted * *p* < 0.05, ** *p* < 0.01, and *** *p* < 0.001 by the Kruskal–Wallis test. Data marked with ns denotes ‘no significance’.

Eye facsimiles were synthesized using three commonly studied biomaterials of gelatin, collagen, and alginate and examined for applied electric field. Data in Figure 5A show that the average electric field measured across four eye facsimiles placed within an extracellular solution of DI water was 16.99 ± 0.92 mV/cm for Alginate-1 (i.e., facsimile synthesized with DI water), 36.93 ± 1.43 mV/cm for collagen, and 15.59 ± 1.49 mV/cm for gelatin. Similarly, the electric field across facsimiles placed within an extracellular solution of DPBS was measured to be 13.19 ± 1.65 mV/cm for Alginate-1, 13.12 ± 2.10 mV/cm for collagen, and 17.10 ± 1.95 mV/cm for gelatin, as per Figure 5B. Average values of electric fields across facsimiles placed within extracellular solutions of DMEM are shown in Figure 5C as 15.92 ± 0.99 mV/cm for Alginate-1, 13.73 ± 0.93 mV/cm for collagen, and 11.73 ± 1.49 mV/cm for gelatin.

In addition, because gelatin and collagen synthesized within a variety of solutions have been previously shown to maintain live cells [34,35], our study examined three different alginate facsimiles synthesized with DI water, DPBS, and DMEM for comparison. As seen in Figure 5D, alginate synthesized in DI water (Alginate-1) exhibited the highest average field of 15.92 ± 0.90 mV/cm, followed by 11.90 ± 1.37 mV/cm in alginate synthesized in DPBS (Alginate-2), and an average electric field of 8.56 ± 0.92 mV/cm synthesized in DMEM (Alginate-3). Based on these data, all subsequent tests utilized alginate synthesized in DMEM to support cell viability (Alginate-3).

### 3.4. Three-Dimensional Confocal Imaging Distinguished Fluorescent Beads and Live Cells Within Facsimiles

**Figure 6.**
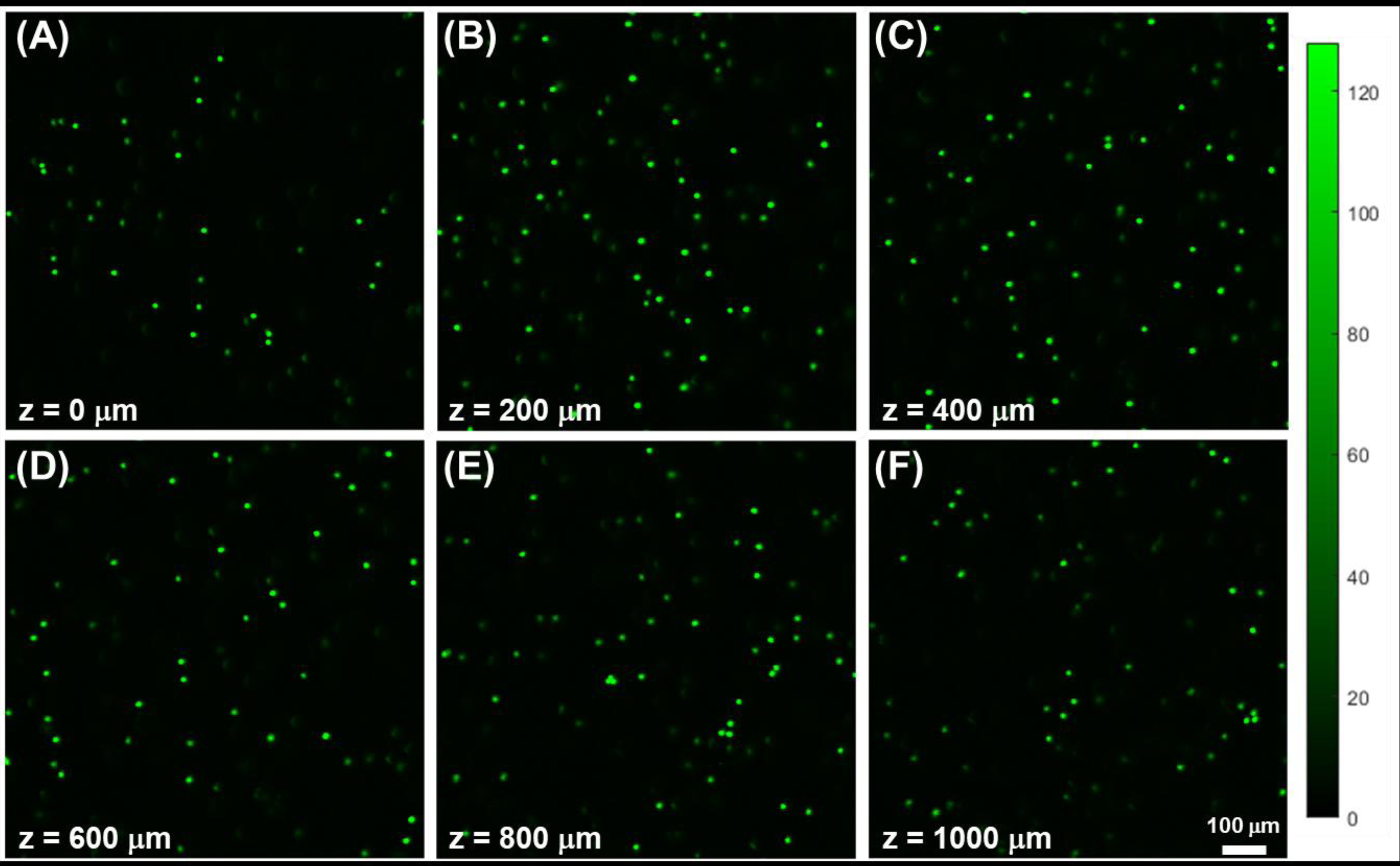
Representative images of 10 μm diameter fluorescent microbeads located at different depths within alginate facsimiles. Microbeads are imaged within different axial (z) planes from (**A**) z = 0 μm (the interface with the cell-rich hydrogel layer), (**B**) z = 200 μm subsurface depth, (**C**) z = 400 μm subsurface depth, (**D**) z = 600 μm subsurface depth, (**E**) z = 800 μm subsurface depth, and (**F**) z = 1000 μm substrate depth. The intensity color scale and 100 μm scale bar apply to all image panels.

The next set of tests synthesized Alginate-3 facsimiles with inert 10 μm diameter fluorescent microbeads (2106G, Phosphorex LLC, Hopkinton, Massachusetts, USA) embedded within the parallel eye device. Beads were seen in confocal images exclusively as individual fluorescent signatures, without wide spacings or aggregation. As seen in Figure 6, distinct bead distributions were imaged at different depths (z-values) within facsimiles, demonstrating the ability to determine the axial location of cell-size objects within the intact facsimiles, within planes from z = 0 μm (i.e., the interface of facsimiles with CRHL) to beyond z = 1000 μm.

**Figure 7.**
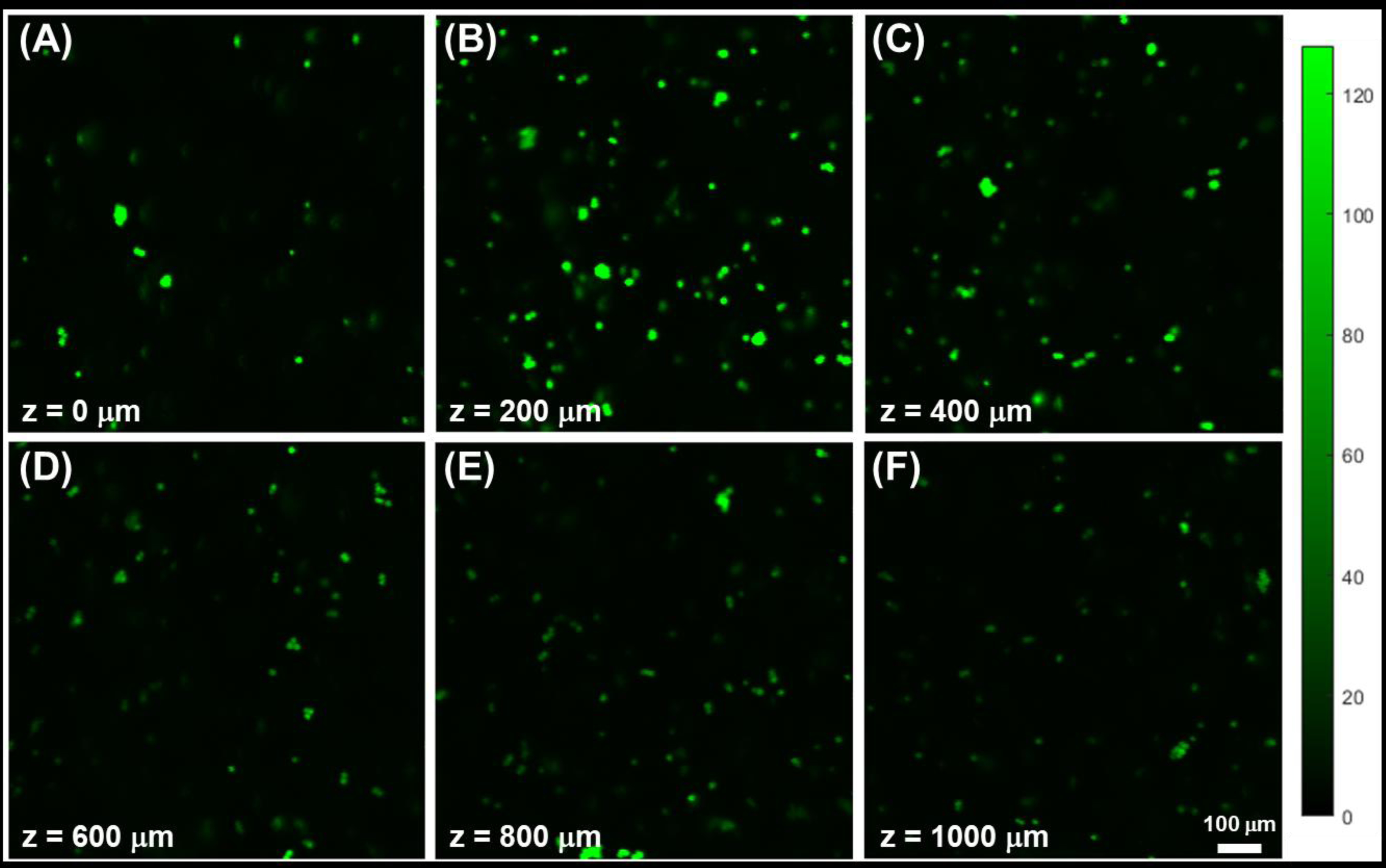
Representative confocal images of fluorescent retinal progenitor cells located at different depths within alginate facsimiles. Progenitors are imaged at different axial (z) planes within whole-eye facsimiles gathered ranging from (**A**) z = 0 μm (interface with the cell-rich hydrogel layer), (**B**) z = 200 μm subsurface depth, (**C**) z = 400 μm subsurface depth, (**D**) z = 600 μm subsurface depth, (**E**) z = 800 μm subsurface depth, and (**F**) z = 1000 μm substrate depth. The intensity color scale and 100 μm scale bar apply to all image panels.

Next, alginate facsimiles were synthesized with fluorescently labeled cells. Confocal images exhibited different average cell diameters and some visible clustering, as seen in Figure 7. As with the fluorescent beads, the confocal microscope was capable of detecting the presence of cells to distances exceeding 1000 μm within the eye facsimile and distinguishing between cells located at different axial (z) planes.

**Figure 8.**
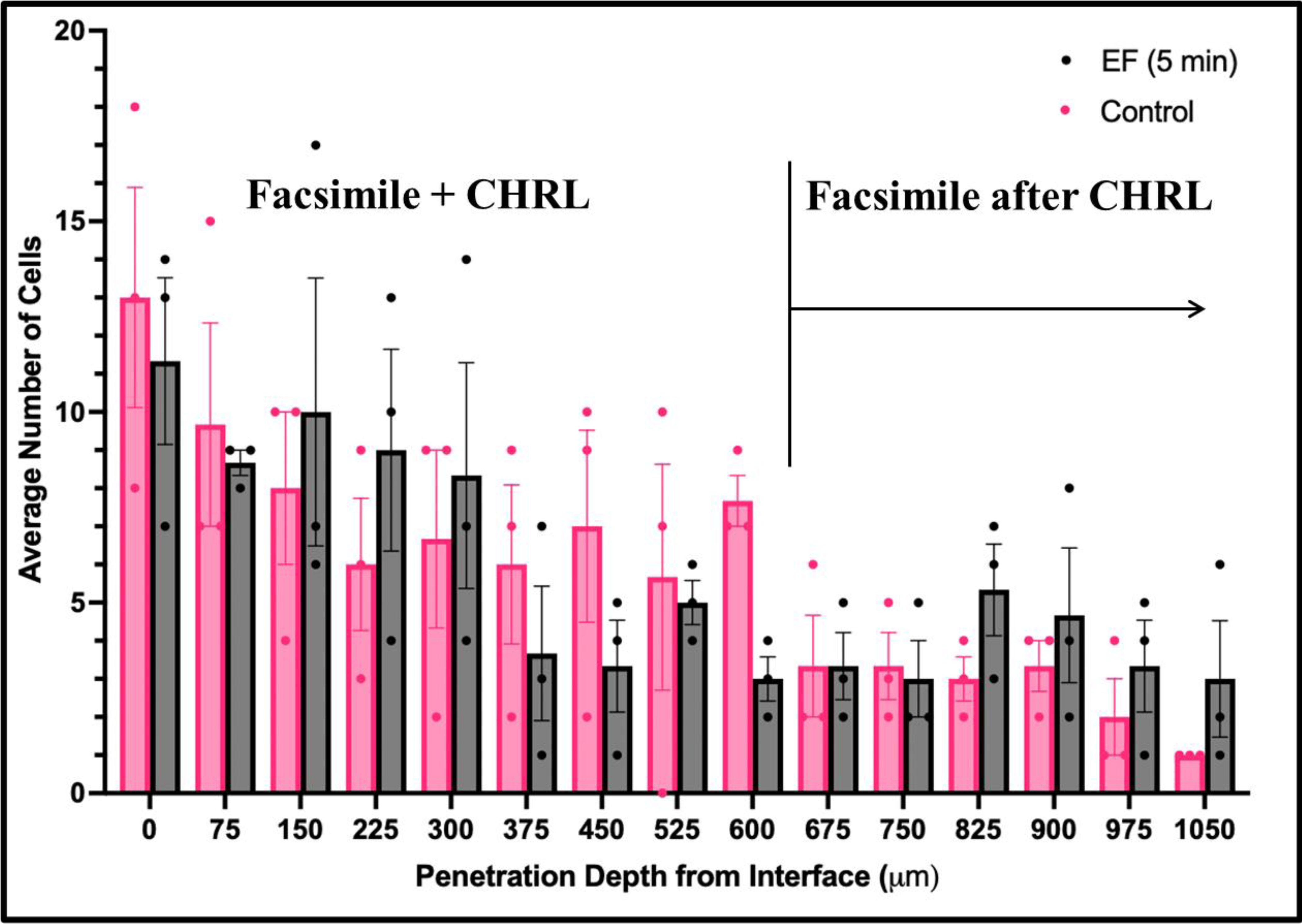
Average numbers of cells electrically stimulated to infiltrate within different depths of alginate eye facsimiles. Data show the average numbers of cells measured per individual z-stack depth within whole-eye facsimiles measured with and without applied electric fields.

The last set of experiments applied an electric field across alginate facsimiles (with no embedded cells) and the adjacent CRHL to examine electrical stimulation on cell infiltration. As shown in Figure 8, optical imaging facilitated the measurement of cell infiltration across different depths within intact facsimiles.

The data in Figure 8 illustrate significant differences in the migration of cells within eye facsimiles with and without EFs, both at specific depths as well as cumulative depths across facsimiles. As shown, the CRHL interfaces with eye facsimiles along the coronal plane for approximately 675 μm from the microscope stage. Measurements from this point onwards illustrate 23 ± 9 cells with EF stimulation compared to 16 ± 4 for the control (no EFs).

## 4. Discussion

Microtechnologies readily enable the development and advancement of regenerative therapies by producing models to recapitulate critical cell processes and environments at the retinal scale [36]. Few in vitro platforms have been applied to cell replacement therapy, where donor stem cells are mixed with biomatrices and transplanted into the host retina to treat adult vision loss. Unfortunately, transplantation studies in animals and clinical trials have reported uneven success, in large part because the primary, but critical, process of cell infiltration out of the delivery matrix and into retinal tissue remains surprisingly understudied. Failure or disorder in this crucial phase may produce biochemical blockades that prevent the appropriate cell differentiation needed for integration with native retinal neurons [37,38]. Our project developed an optical–parallel eye system to evaluate the electrically driven infiltration of cells within 3D eye facsimiles made of spherical biomaterials. This novel paired system enables rapid, in situ visualization of cell penetration depths within eye facsimiles in response to external applied electric fields (EFs) to facilitate the development of migration-targeted approaches to transplantation. Importantly, optical imaging facilitates rapid evaluation of applied fields without time-intensive cryo-sectioning. This enables the selection of the frequency, duration, and strength of applied fields to achieve desired migration distances. These contributions complement existing biological studies, as molecular and cellular determinants of migratory behavior can now be incorporated in the derivation of specialized replacement cells.

The parallel eye system combines eye facsimiles with hydrogels containing replacement cells within a support plate produced in a similar configuration to 48-well plates of benchtop biology (Figure 3). An EF is applied across the system to stimulate cell infiltration out of the hydrogel and into the facsimile by applying a potential difference. The initial tests using a constant DC voltage of 0.3V were used to generate an electric field of 100 mV/cm for 10 min to match previous studies of electric fields in retinal progenitors [18]. However, the resistor model of the parallel eye device enables variation in DC voltage to generate variable electric fields for variable time periods. Moreover, commercial data acquisition devices can generate voltage differences from 0 to 10V for continuous time points of 0 to 12 h as desired. Our platform thereby enables a diversity of investigations into the effects of applied electric fields to support in vivo applications for transplantation.

A novel and critical component of the parallel eye system is its integration with scanning confocal microscopy to perform rapid, non-destructive analysis of changes in penetration depth as a function of EF. Surprisingly, EFs remain underexamined in many regenerative therapies, despite being therapeutically applied across the nervous system with many positive vision outcomes. These include post-injury and disease [39], across specific ocular components [14], as well as via cortical and retinal implants [40]. Our group is among the first to apply EFs to aid therapeutic outcomes by guiding the 3D infiltration of replacement cells, i.e., the explicit first step in the transplantation process. This engineering direction is key to advancing vision treatment for two reasons. First, the migration of replacement cells within convoluted and interconnected pores of 3D structures is more representative of in vivo processes than microscale studies of single or collective cell migration [41]. Second, the clinical translation of ocular EFs to aid therapeutic outcomes is most applicable across whole eyes in tandem with commercial electrodes already in clinical use [42]. The parallel eye system is thereby developed with the ultimate goal of evaluating in vivo therapeutic EFs in future studies as currently used in trans-orbital and intraocular deep stimulation to treat eye disease.

Eye facsimiles of gelatin, collagen, and alginate were evaluated for our system because each material has been studied extensively for applications in drug delivery [43,44], implants [44,45], grafts [46,47], and biomaterial substitutes [48,49] for diverse ocular components. Tests measured the abilities of each biomaterial to sustain an applied electric field across its porous 3D structure when surrounded by extracellular solutions of different ionic strengths (Figure 5). The surrounding extracellular solution is significant because of well-known ionic influences on electrostatic interactions between macromolecules and cells, toxicity, and viability [51–52]. Moreover, although an extracellular solution of lower ionic strength facilitates a stronger EF influence on cells, the sustained EF may impact fundamental motility processes via the rearrangement of microtubules [53]. DMEM was selected as the extracellular solution for testing because it produced the lowest EF variability across all biomaterials. Alginate was similarly selected over gelatin and collagen for its consistent electrical field. We note that alginate was also desirable for infiltration study because its viscoelastic properties are readily altered using known crosslinking agents, which may become significant to the migration of replacement cells [54,55]. Additional tests confirmed that its pores maintained consistent electric fields in the surrounding extracellular solution of DMEM (Figure 5), while computational simulations validated that Alginate-3 facsimiles supported EF-induced migration of a model ionic molecule (Figure 4). The results predicted that EF across the facsimiles induced molecular migration across its full diameter in less than 2 h, which provided an important minimum time point for the testing.

The parallel eye device then combined Alginate-3 facsimiles with the cell-rich hydrogel layer (CRHL) for EF testing. Control studies first validated the use of confocal imaging by performing a depth-resolved analysis of cell-mimicking fluorescent beads distributed within eye facsimiles (Figure 7). The data illustrated the ability of the confocal instrument to resolve individual microbeads both laterally (within an image) and axially (corresponding to depth within the facsimile), supporting the potential of the method for quantifying cell distribution within facsimiles. Low-magnification imaging (10×) allowed imaging over a relatively large field of view (1 mm × 1 mm) while still resolving sufficient detail. When cultured cells were fluorescently labeled and imaged in eye facsimiles using the same instrument, cells with different semi-spherical and elongated morphologies were again resolved at different penetration depths extending to approximately 1000 μm (Figure 8). These data demonstrate that confocal fluorescence imaging is sufficiently sensitive to detect and resolve signals from cells labeled with a commonly used fluorophore (i.e., Calcein AM) at depths exceeding the relevant length scales in clinical practice.

The final set of experiments applied our paired microscale–optical system to measure differences in the average numbers of cells at different penetration depths of eye facsimiles (Figure 8). The data illustrated significant differences in the migration of cells within eye facsimiles with and without EFs, both at specific depths as well as cumulative across facsimiles. All solutions were kept sterile, and after electric field application, the device was left in an incubator kept at 37C and 5% CO_2_ for six hours. We note that cell viability is readily increased to 1–2 days when an incubated stage is used. Further, the parallel eye system facilitates the testing of up to four facsimiles at once with the potential for variable electric fields. However, new hydrogel resistors must be synthesized with every use due to shrinkage. Future testing will optimize the positioning of system components to more accurately evaluate cell infiltration within sagittal, coronal, and axial planes of eye facsimiles. These results are highly significant to the development of platforms to advance transplantation therapy. EFs hold exciting potential to stimulate and/or guide the migration of replacement cells within host tissue but have lacked consolidated platforms for rapid testing. This study has developed a model to advance the development of electrical-based therapies for cell transplantation. Our data show that the application of small electric fields for short durations causes large increases in the migration of transplantable cells within eye facsimiles. These data allow future studies to apply electric fields to enucleated eyes of different animals to more appropriately study human disease to ultimately develop therapies for in vivo testing.

## 5. Conclusions

The parallel eye system enables the study of replacement cell infiltration within biomaterial-based eye facsimiles. The system can be adapted to apply multiple external fields, use facsimiles of different biomaterials and enucleated eyes from different animal models, and examine behaviors of specialized replacement cells. The use of confocal optical microscopy to assess outcomes offers an exciting translational pathway, as confocal scanning laser ophthalmoscopy is routinely used in clinical practice. The system thereby provides a platform to advance new strategies aimed at improving the outcomes of transplantation therapy in the adult retina and in the nervous system more broadly.

## Supplementary Material

Supplemental Figure 1

Supplementary Materials:

## Data Availability

The original contributions presented in this study are included in the article/supplementary material. Further inquiries can be directed to the corresponding author(s).
