# Supplementary figures and images for: A Microscale–Optical Interface to Examine Electric Field-Induced Cell Motility Within Whole-Eye Facsimiles
Source: Micro (Basel). Author manuscript; Available in PMC 2025 Jun 30. (PMC12208019; doi:10.3390/micro5010010)

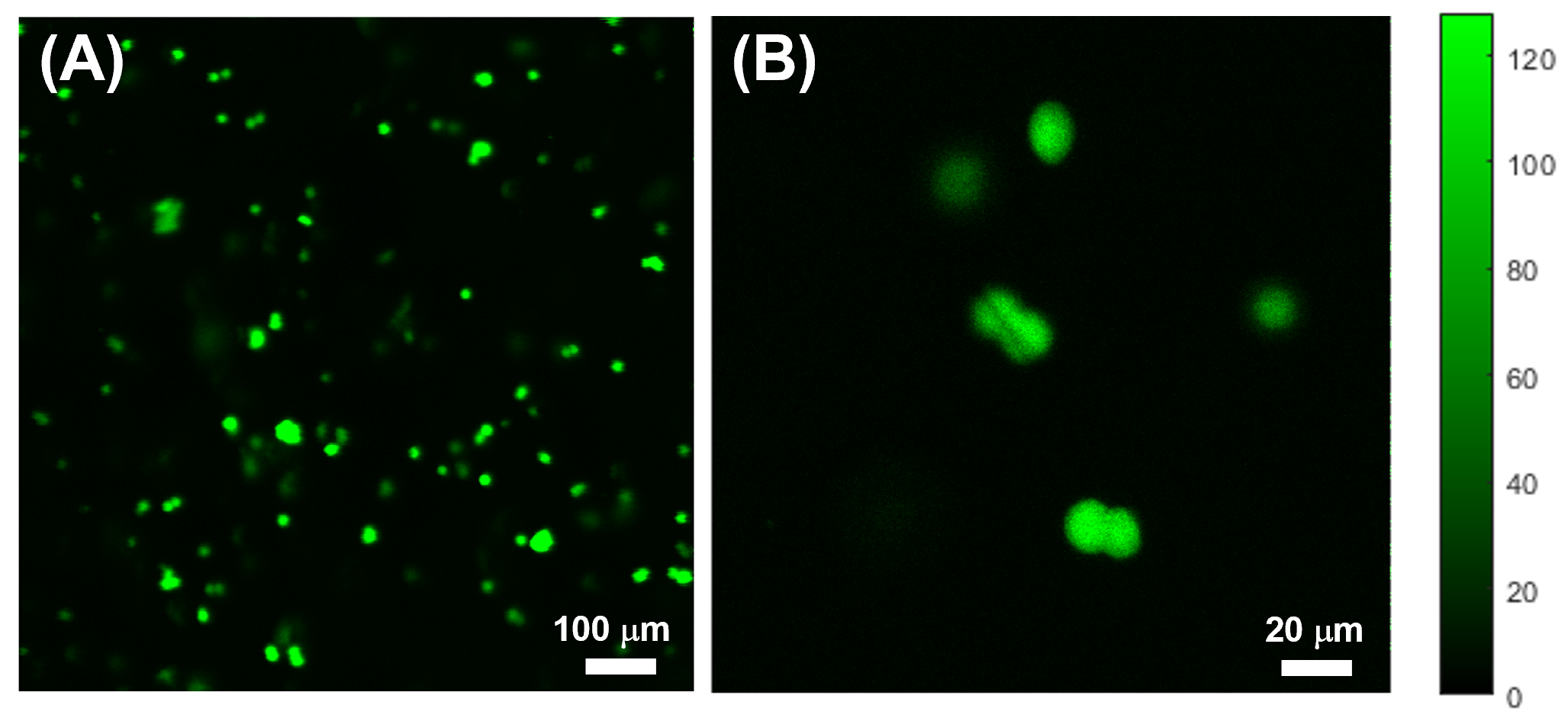

Supplement: Supplemental Figure 1 [file NIHMS2077670-supplement-Supplemental_Figure_1.tif]
